# New Undisputed Evidence and Strategy for Enhanced Lattice‐Oxygen Participation of Perovskite Electrocatalyst through Cation Deficiency Manipulation

**DOI:** 10.1002/advs.202200530

**Published:** 2022-03-20

**Authors:** Xiaomin Xu, Yangli Pan, Yijun Zhong, Chenliang Shi, Daqin Guan, Lei Ge, Zhiwei Hu, Yi‐Ying Chin, Hong‐Ji Lin, Chien‐Te Chen, Hao Wang, San Ping Jiang, Zongping Shao

**Affiliations:** ^1^ WA School of Mines: Minerals, Energy and Chemical Engineering (WASM‐MECE) Curtin University Perth WA 6102 Australia; ^2^ Centre for Future Materials University of Southern Queensland Springfield Central QLD 4300 Australia; ^3^ State Key Laboratory of Materials‐Oriented Chemical Engineering College of Chemical Engineering Nanjing Tech University Nanjing 211800 China; ^4^ Department of Building and Real Estate Research Institute for Sustainable Urban Development (RISUD) and Research Institute for Smart Energy (RISE) The Hong Kong Polytechnic University Hung Hom Kowloon Hong Kong 999077 China; ^5^ School of Chemical Engineering The University of Queensland Brisbane QLD 4072 Australia; ^6^ Max Planck Institute for Chemical Physics of Solids Nöthnitzer Str. 40 Dresden 01187 Germany; ^7^ Department of Physics National Chung Cheng University Min‐Hsiung Chiayi 62102 Taiwan; ^8^ National Synchrotron Radiation Research Center Hsinchu 30076 Taiwan

**Keywords:** cation deficiency, lattice‐oxygen participation, oxygen evolution reaction, perovskites, water splitting, Zn–air batteries

## Abstract

Oxygen evolution reaction (OER) is a key half‐reaction in many electrochemical transformations, and efficient electrocatalysts are critical to improve its kinetics which is typically sluggish due to its multielectron‐transfer nature. Perovskite oxides are a popular category of OER catalysts, while their activity remains insufficient under the conventional adsorbate evolution reaction scheme where scaling relations limit activity enhancement. The lattice oxygen‐mediated mechanism (LOM) has been recently reported to overcome such scaling relations and boost the OER catalysis over several doped perovskite catalysts. However, direct evidence supporting the LOM participation is still very little because the doping strategy applied would introduce additional active sites that may mask the real reaction mechanism. Herein, a dopant‐free, cation deficiency manipulation strategy to tailor the bulk diffusion properties of perovskites without affecting their surface properties is reported, providing a perfect platform for studying the contribution of LOM to OER catalysis. Further optimizing the A‐site deficiency achieves a perovskite candidate with excellent intrinsic OER activity, which also demonstrates outstanding performance in rechargeable Zn–air batteries and water electrolyzers. These findings not only corroborate the key role of LOM in OER electrocatalysis, but also provide an effective way for the rational design of better catalyst materials for clean energy technologies.

## Graphical abstract

A dopant‐free, cation‐deficiency manipulation strategy is reported to regulate the bulk oxygen‐ion diffusion properties of perovskites without altering their surface properties, offering a perfect platform to understand the role of lattice‐oxygen participation in oxygen evolution reaction electrocatalysis. Optimizing the cation deficiency level identifies a perovskite candidate with excellent catalytic activity applicable in Zn–air batteries and water electrolyzers.

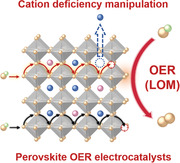

## 1 Introduction

Oxygen evolution reaction (OER), also referred to as water oxidation, is widely recognized as an important electrode reaction of both fundamental and practical interest.^[^ ^1^ ^]^ For instance, the OER dominates largely the overall efficiency of many electrochemical energy conversion devices such as rechargeable metal‐air batteries and water electrolyzers.^[^ ^2^, ^3^, ^4^, ^5^ ^]^ However, due to its complex four‐electron transfer, the OER process suffers from intrinsically poor kinetics, which often requires a considerable overpotential to realize moderate reaction rates, thus limiting the overall efficiency of the related energy devices.^[^ ^1^, ^2^, ^3^, ^4^, ^5^ ^]^ To lower this kinetic barrier, electrocatalysts containing noble metals, like iridium oxide (IrO_2_) and ruthenium oxide (RuO_2_), are often employed,^[^ ^6^ ^]^ which show one of the best OER performances, but their larger‐scale deployment is hindered by their low abundance and high cost. Therefore, tremendous efforts have been made to develop non‐noble metal‐based alternatives that are competitively efficient.^[^ ^7^, ^8^, ^9^ ^]^

Among various candidates, ABO_3_‐type perovskite oxides, where A and B is respectively a rare‐earth or alkaline‐earth metal and a transition metal, are promising OER electrocatalysts thanks to their low cost, easy synthesis, and high catalytic activity.^[^ ^10^, ^11^, ^12^ ^]^ In particular, by leveraging their compositional flexibility, which allows elemental doping or substitution at all the A‐, B‐, and O‐sites, the crystalline structure and electronic structure of perovskite oxides can be fine‐tuned, providing plenty of room for the rational design of better OER catalysts. Indeed, the last decade has witnessed a surge in exploring a plethora of perovskite compositions as electrocatalysts for driving the OER in an alkaline medium (4OH^−^ → O_2_ + 2H_2_O + 4e^−^).^[^ ^13^, ^14^, ^15^, ^16^, ^17^, ^18^, ^19^ ^]^ For instance, Shao‐Horn et al. screened more than ten perovskite oxides and discovered a volcano‐shaped relationship between the OER activity and the *e*_g_ occupancy of the B‐site transition metal, whereby Ba_0.5_Sr_0.5_Co_0.8_Fe_0.2_O_3−_ *_*δ*_* (BSCF) with *e*_g_ ≈ 1 gives a high catalytic activity.^[^ ^13^ ^]^ More importantly, this compositional versatility of perovskite oxides can offer a great platform for studying the reaction mechanisms underlying the OER. For example, with the increasing doping of Sr into the La_1−_ *_x_* Sr*_x_* CoO_3−_ *_*δ*_* system, the mechanism to catalyze the OER has been recently demonstrated to transition from a traditional adsorbate evolution reaction mechanism (AEM) to a new, lattice oxygen‐mediated mechanism (LOM).^[^ ^20^, ^21^, ^22^ ^]^ Our group has also offered direct evidence of the lattice oxygen involvement in the OER catalysis through the design of a Si‐incorporated SrCo_1−_ *_y_* Si*_y_* O_3−_ *_*δ*_* system.^[^ ^23^ ^]^ The operation of the LOM was claimed to be capable of bypassing the scaling relation‐based limitations in the AEM,^[^ ^14^ ^]^ thus providing another playground to construct better OER catalysts. Despite success in the doping strategy, one challenge persists for these doped perovskites in that the incorporation of foreign elements can have an impact on the phase structure and/or the amount of B‐site transition metal atoms, significantly increasing the degrees of freedom for the electronic structure tuning and hence complicating the understanding of the structure‐property relationships of perovskite catalysts.

Ideally, the ABO_3_ perovskites should have an A‐site to B‐site cation ratio of unity. However, it was found that for some perovskites the structural integrity can be still maintained when a proportion of the A‐ or B‐site cations become deficient.^[^ ^24^ ^]^ Generally, there are two types of cation‐deficient perovskites, namely the A‐site cation‐deficient perovskites and the B‐site cation‐deficient perovskites (denoted A_1−_ *_z_* BO_3_ and AB_1−_ *_z_* O_3_, where *z* is the deficiency level), with the former being energetically more favorable.^[^ ^25^ ^]^ Unlike elemental doping, the creation of cation deficiency is expected to affect the physicochemical properties of the perovskite oxides without compromising or introducing additional elements into the pristine crystalline structure. Over the years, there have been increasing interests in developing cation‐deficient perovskites for diverse energy‐related research areas, including solid oxide electrochemical cells,^[^ ^26^, ^27^, ^28^ ^]^ dye‐sensitized solar cells,^[^ ^29^ ^]^ and electrocatalysis.^[^ ^30^, ^31^, ^32^, ^33^ ^]^ For example, introducing A‐site La‐deficiency in the LaFeO_3_ *_−*δ*_* perovskite was found to bring additional oxygen vacancies as well as a certain amount of Fe^4+^ species (with *e*_g_ = 1), both contributing to the OER catalysis under the AEM scheme. Similar endeavors have been undertaken in Mn‐, Co‐, and Ni‐based perovskites.^[^ ^31^, ^32^, ^33^ ^]^ However, to date, insights into how the introduction of cation deficiency can impact the LOM‐based perovskite catalysts are still lacking.

In this work, we demonstrate that the tailoring of A‐site cation deficiency can be used to create different oxygen diffusion rates of the perovskite oxide bulk, thus providing a perfect platform for studying the role of lattice‐oxygen participation in the OER, and that such cation deficiency manipulation can be further leveraged to develop new outstanding perovskite‐based electrocatalysts for OER. By tuning the deficiency level, a cation‐deficient perovskite with optimized OER activity is facilely obtained, which outperforms the IrO_2_ benchmark when tested in energy devices including rechargeable zinc–air batteries and water electrolyzers, showcasing the great promise for practical use. These results not only highlight the feasibility of introducing A‐site cation deficiency for enhancing the OER catalysis on perovskite oxide catalysts that utilize the LOM mechanism, but also underscore the fundamental origin of this activity enhancement, which can have enormous implications for the design of better catalyst materials for clean energy technologies.

## 2 Results and Discussion

### 2.1 A‐Site Cation Deficiency Manipulation

We selected a La‐Sr‐Co‐Fe‐O perovskite oxide system, a prototype known for its high oxygen‐evolving performance,^[^ ^34^, ^35^, ^36^, ^37^ ^]^ to investigate the effect of cation deficiency on the electrocatalytic OER. To tailor the catalytic performance of a perovskite catalyst through manipulating the cation deficiency, it is important to know the maximum deficiency that the perovskite can tolerate since the phase transition or the formation of secondary phases would introduce additional factors for affecting the catalytic performance, hence masking the exact contribution of lattice‐oxygen participation in the OER process. It is well known that the A‐site cation deficiency is energetically more favorable than the B‐site cation deficiency, we, therefore, on purpose introduced different levels of Sr deficiency in a La‐Sr‐Co‐Fe‐O series with the nominal chemical formula of La_1/3_Sr(_2−3_ *_z_*)_/3_Co_0.5_Fe_0.5_O_3−_ *_*δ*_*, or LaSr(_2−3_ *_z_*)Co_1.5_Fe_1.5_O_9−3_ *_*δ*_* (abbreviated as LaSr2−3*z*), where 3*z* = 0.00, 0.05, 0.10, 0.15, and 0.20, denoted LaSr2.00, LaSr1.95, LaSr1.90, LaSr1.85, and LaSr1.80, respectively. A facile sol‐gel approach (see Supporting Information for more details) was adopted to ensure the atomic‐level mixing of the raw materials, thus the obtained phases represent the thermodynamically stable phases.

**Figure 1:**
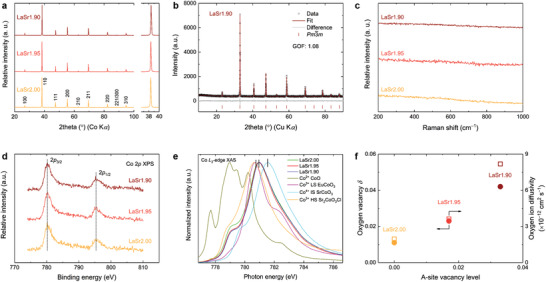
Physicochemical characterizations of Sr‐deficient perovskites. a) XRD data of LaSr2.00, LaSr1.95, and LaSr1.90 (collected using a Co K*α* source). b) Rietveld refinement result using the XRD data of LaSr1.90 (collected using a Cu K*α* source). GOF: goodness of fit. c–e) Raman spectra (c), Co 2*p* XPS spectra (d), and Co *L*_3_‐edge XAS spectra (e) of LaSr2.00, LaSr1.95, and LaSr1.90. Reference data for Co *L*_3_‐edge XAS spectra were taken from EuCoO_3_ for LS (low‐spin state) Co^3+^,^[^ ^46^ ^]^ SrCoO_3_ for IS (intermediate‐spin state) Co^4+^,^[^ ^47^ ^]^ and Sr_2_CoO_3_Cl for HS (high‐spin state) Co^3+^.^[^ ^46^, ^48^ ^]^ f) Oxygen vacancy content (*δ*) and oxygen ion diffusivity as a function of A‐site Sr‐deficiency level.

**Figure** **1**a shows the room‐temperature X‐ray diffraction (XRD) data of the various samples after the calcination at 1050 °C in air. As expected, the cation stoichiometric sample LaSr2.00 took a single cubic perovskite phase with space group *Pm* $\bar{3}$ *m*. For both LaSr1.95 and LaSr1.90, the same single cubic perovskite structure was demonstrated, as exemplified by the Rietveld refinement analysis of the LaSr1.90 sample (Figure 1b). Interestingly, the corresponding diffraction peaks overlap perfectly with the LaSr2.00 sample, suggesting that the LaSr2−3*z* perovskite can tolerate a certain degree of A‐site cation deficiency while the lattice parameters are almost unchanged. With the further increase of 3*z* to 0.15, the formation of additional phases related to transition metal‐based oxides was observed (Figure S1, Supporting Information). The presence of such an impurity phase became more obvious when the nominal A‐site deficiency of 3*z* reached 0.20 (Figure S2, Supporting Information). It suggests that the maximum A‐site Sr‐deficiency level in the LaSr2−3*z* series is between *z* = 0.033 and 0.050 (i.e., 3*z* = 0.10*–*0.15). While larger deficiency levels were found in several other perovskite systems, such as La_1−_ *_z_* FeO_3_ *_−*δ*_* (*z =* 0.10),^[^ ^30^ ^]^ (Ba_0.5_Sr_0.5_)_1−_ *_z_* Co_0.8_Fe_0.2_O_3−_ *_*δ*_* (*z* = 0.15),^[^ ^38^ ^]^ and (La,Sr)_1−_ *_z_* (Ti,M)O_3−_ *_*δ*_* (*z* = 0.20 for M = Mn, Fe, Ni, and Cu),^[^ ^39^ ^]^ we note that the low tolerance limit for the Sr‐deficiency in our case is likely associated with the intrinsic chemistry of the La‐Sr‐Co‐Fe‐O series, which was also observed for a similar perovskite chemistry reported earlier (for instance, La_0.6_Sr_0.4−_ *_z_* Co_0.2_Fe_0.8_O_3−_ *_*δ*_* with *z* = 0.025).^[^ ^40^ ^]^ The successful formation of single cubic perovskite phase for the LaSr1.95 and LaSr1.90 samples was further corroborated by Raman spectroscopy. As shown in Figure 1c, similar to LaSr2.00, there is an absence of Raman active bands in both LaSr1.95 and LaSr1.90 samples, which is a good indication of the cubic perovskite structure.^[^ ^41^ ^]^ We also performed high‐resolution transmission electron microscopy to ascertain the crystalline structure of the sample LaSr1.90. As displayed in Figure S3, Supporting Information, lattice fringe distances of 0.38 and 0.27 nm were observed, matching perfectly with the (001) and ($\bar{1}10$) reflections in the corresponding XRD data. The fast Fourier transformed pattern along the [110] zone axis further confirms the cubic phase structure of LaSr1.90 (inset of Figure S3, Supporting Information).

Generally, introducing Sr‐deficiency would cause a change in cation stoichiometry and hence an imbalance in net charge, which needs to be compensated to maintain the overall electrical neutrality within the perovskite system. Such compensation may be realized through either the increase of the oxidation state of the B‐site cations and/or the generation of additional oxygen vacancies.^[^ ^25^ ^]^ It is well known that B‐site cations play an important role in the electrocatalytic OER based on the conventional AEM scheme, while the same cation with different oxidation states may show a fairly different catalytic activity. To probe the possible electronic structure change due to the introduction of Sr‐deficiency, X‐ray photoelectron spectroscopy (XPS) as a surface‐sensitive technique was first implemented,^[^ ^41^ ^]^ with the results for the cobalt element shown in Figure 1d. The Co 2*p* XPS spectra of the LaSr2−3*z* series (3*z* = 0*–*0.10) exhibited nearly identical peak positions and shapes, implying insignificant modifications in the surface oxidation state of cobalt. To glean the near‐surface information of the electronic structure of perovskite catalysts, we further performed soft X‐ray absorption spectroscopy (XAS) in the total electron yield (TEY) mode, which is highly sensitive to the valence^[^ ^42^, ^43^ ^]^ and spin states^[^ ^44^, ^45^ ^]^ of the transition metal cations. Figure 1e presents the Co *L*_3_‐edge XAS spectra of LaSr2.00, LaSr1.95, and LaSr1.90, along with those of reference samples including CoO (for Co^2+^), EuCoO_3_ (for low‐spin state Co^3+^),^[^ ^46^ ^]^ SrCoO_3_ (for intermediate‐spin state Co^4+^),^[^ ^47^ ^]^ and Sr_2_CoO_3_Cl (for high‐spin state Co^3+^ in pyramidal local coordination).^[^ ^46^, ^48^ ^]^ The peaks of these XAS spectra shift toward higher photon energy with increasing Covalence, which, together with the almost unchanged peak position, suggests that the surface oxidation state of the Co cation was in the range of +3 to +4 and was hardly affected by the introduction of a small amount of Sr deficiency. Judging from the relative peak positions and spectral weight changes, the Co cations in LaSr2.00, LaSr1.95, and LaSr1.90 had a valence state of approximately +3.3 to +3.4 (Figure 1e). Note that such an oxidation state of Co was previously reported to be optimal for the OER catalysis over Co‐based oxide electrocatalysts.^[^ ^36^, ^49^, ^50^ ^]^ The surface electronic state of iron was also found to be similar for the various LaSr2−3*z* samples, as evidenced from the analogous features of the Fe *L*_3_‐edge XAS spectra (Figure S4, Supporting Information).

Given the unchanged B‐site Co/Fe oxidation state, it is clear that the compensation for the charge imbalance caused by the introduction of A‐site Sr‐deficiency was solely realized through the creation of oxygen vacancies, likely following a so‐called A‐site‐vacancy mechanism,^[^ ^51^ ^]^ as expressed by the below Kröger‐Vink notation:

(1) $${\mathrm{Sr}}_{\mathrm{Sr}}^{X}+O_{O}^{X}\to V_{\mathrm{Sr}}^{{}^{\prime \prime }}+V_{O}^{••}+\mathrm{SrO}$$

where ${\mathrm{Sr}}_{\mathrm{Sr}}^{X}$ represents a Sr ion sitting on a Sr sublattice site with a neutral charge, $O_{O}^{X}$ represents an O ion sitting on an O sublattice site with a neutral charge, $V_{\mathrm{Sr}}^{{}^{\prime \prime }}$ represents a Sr vacancy with a double negative charge, and $V_{O}^{••}$ represents an O vacancy with a double positive charge. To support the increase of oxygen vacancies through introducing A‐site cation deficiency, the oxygen vacancy content of the LaSr2.00, LaSr1.95, and LaSr1.90 samples was measured by the standard chemical titration method with the experimental procedures detailed in the Supporting Information. Indeed, an increasing number of oxygen vacancies was observed with increasing Sr‐deficiency level (Figure 1f and Table S1, Supporting Information). From the obtained oxygen vacancy concentrations, the bulk average B‐site oxidation state was calculated to be almost invariant regardless of the Sr stoichiometry (Table S1, Supporting Information), consistent with what was observed for the surface as mentioned above.

As we know, oxygen vacancies are the charge carriers for oxygen ions.^[^ ^52^ ^]^ An increase in the oxygen vacancy concentration may lead to an increase in oxygen ion diffusion, and an increase in OER performance is then expected if the LOM mechanism is operative during the OER process. The oxygen ion diffusion coefficient of the LaSr2.00, LaSr1.95, and LaSr1.90 was measured using combined electrochemical techniques of cyclic voltammetry (CV) and chronoamperometry (CA) (see Supporting Information for more details). Shown in Figure S5, Supporting Information are the CV data recorded in an argon‐saturated 6 M KOH alkaline solution, where redox features characteristic of the intercalation and de‐intercalation of oxygen ions were observed. As expected, the Sr‐deficient samples exhibited larger current densities relative to the pristine LaSr2.00, indicating a greater tendency for oxygen intercalation. Following the oxygen intercalation experiments, the oxygen ion diffusion coefficient was obtained using the CA technique (Figure S6, Supporting Information) by applying a previously established bounded 3D diffusion model.^[^ ^53^ ^]^ The oxygen ion diffusivity for LaSr2.00 was calculated to be 1.96 × 10^−12^ cm^2^ s^−1^, consistent with the previously reported results,^[^ ^36^ ^]^ which increased to 3.62 and 8.18 × 10^−12^ cm^2^ s^−1^ for the Sr‐deficient LaSr1.95 and LaSr1.90, respectively (Table S2, Supporting Information), correlating well with the increased amount of oxygen vacancy (Figure 1f).

The above analysis suggests that the proper manipulation of the Sr cation deficiency in the LaSr2−3*z* series can maintain the perovskite structure as well as the chemical state of the transition metal cations while introducing additional oxygen vacancies into the oxide lattice to result in increased oxygen diffusion rate. Since no other foreign element is introduced through manipulating the A‐site cation deficiency, the A‐site cation‐deficient LaSr2−3*z* then provides a perfect platform for the investigation of the role of lattice‐oxygen participation in the OER electrocatalysis.

### 2.2 Electrocatalytic OER Performance and Mechanism

**Figure 2:**
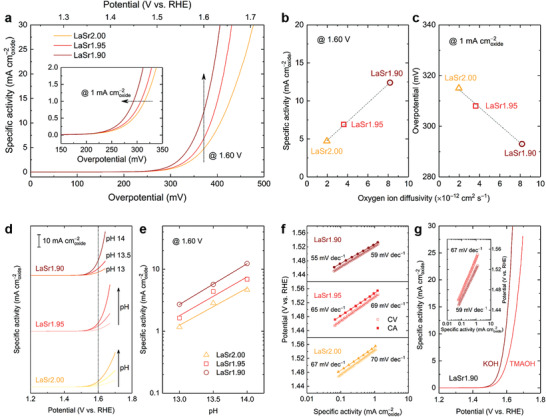
Electrocatalytic OER performance and mechanism studies of Sr‐deficient perovskites. a) Polarization curves of LaSr2.00, LaSr1.95, and LaSr1.90 in a 1 M KOH electrolyte. Inset shows a magnification near the low‐overpotential region. b–c) Correlation of oxygen ion diffusivity with the intrinsic OER activity in 1 M KOH in terms of (b) the current density at a select potential of 1.60 V versus RHE and (c) the overpotential at a given current density of 1 mA ${\mathrm{cm}}_{\mathrm{oxide}}^{-2}$. d) Intrinsic OER activity of LaSr2.00, LaSr1.95, and LaSr1.90 under various pH conditions. e) Relationship between the intrinsic OER activity at 1.60 V versus RHE and the electrolyte pH. f) Comparison of Tafel plots using data from both CV and CA measurements tested in 1 M KOH. g) Comparison of OER activity of LaSr1.90 in 1 M KOH and 1 M TMAOH electrolytes. Inset shows the corresponding Tafel plots.

To examine the potential role of LOM in the OER electrocatalysis, the catalytic activity of the various as‐synthesized LaSr2−3*z* oxides toward the alkaline OER was first assessed using a rotating disk electrode (RDE)‐based methodology.^[^ ^15^, ^54^ ^]^ **Figure** **2**a shows the polarization curves in a 1 M KOH electrolyte, where currents were normalized to the Brunauer‐Emmett‐Teller surface area (Table S3, Supporting Information) of LaSr2.00, LaSr1.95, and LaSr1.90 to obtain specific activity (in mA ${\mathrm{cm}}_{\mathrm{oxide}}^{-2}$) as a metric to compare their intrinsic OER activity.^[^ ^13^ ^]^ On introducing Sr deficiency, both samples (i.e., LaSr1.95 and LaSr1.90) showed improved OER performance compared to the cation stoichiometric LaSr2.00 catalyst, as evidenced from a shift in the (over)potential toward more negative values (Figure 2a inset). Notably, the OER activity follows the order of LaSr2.00 < LaSr1.95 < LaSr1.90, indicating that an increase in the Sr‐deficiency level favors the OER catalysis. The monotonic increase of OER activity with the Sr‐deficiency amount is more obviously demonstrated in Figure S7, Supporting Information. To achieve a specific activity of 1 mA ${\mathrm{cm}}_{\mathrm{oxide}}^{-2}$, the overpotential needed for the pristine LaSr2.00 is 315 mV, which dropped to 308 mV for LaSr1.95 and 293 mV for LaSr1.90. In addition, at a select potential of 1.60 V versus the reversible hydrogen electrode (RHE), the specific activity showed an enhancement of 1.5‐fold and 2.6‐fold for LaSr1.95 (6.9 mA ${\mathrm{cm}}_{\mathrm{oxide}}^{-2}$) and LaSr1.90 (12.4 mA ${\mathrm{cm}}_{\mathrm{oxide}}^{-2}$), respectively, relative to LaSr2.00 (4.7 mA ${\mathrm{cm}}_{\mathrm{oxide}}^{-2}$). These observations suggest that introducing A‐site Sr‐deficiency is beneficial to the OER catalysis. Remarkably, the intrinsic OER activity was found to have a strong correlation with the oxygen ion diffusivity, presented in Figure 2b,c. Since the introduction of the proper A‐site cation deficiency only leads to a change in the oxygen vacancy content, and consequently an increase in the oxygen ion diffusion rate, it thus provides an undisputed evidence that the LOM plays an essential role in the OER electrocatalysis.

For the LOM mechanism, it was reported that a strong relation of the catalytic activity to the pH value of the alkaline electrolyte is experienced.^[^ ^22^, ^23^ ^]^ Here we further investigated the catalytic performance of LaSr2.00, LaSr1.95, and LaSr1.90 under different pH conditions. As shown in Figure 2d, for all three samples, increasing the pH from 13 to 14 resulted in enhancements of OER currents on the same RHE reference electrode scale. Such a pH dependence of the OER kinetics is a good indicator of the participation of lattice oxygen during the OER, following the LOM reaction mechanism.^[^ ^22^, ^23^ ^]^ In this LOM pathway, one of the concerted proton‐electron transfer steps in the traditional AEM mechanism becomes decoupled, in which the proton transfer could be the rate‐determining step.^[^ ^55^ ^]^ When further compared at 1.60 V versus RHE, the logarithm of specific activity was found to show a linear relationship with the electrolyte pH (Figure 2e), based on which the proton reaction order on the RHE scale (*ρ*_RHE_) was extracted from the slopes to be 0.60, 0.63, and 0.66 for LaSr2.00, LaSr1.95, and LaSr1.90, respectively, using the below equation:^[^ ^55^ ^]^

(2) $$\rho_{\mathrm{RHE}}={\left(\frac{\partial \log\left(i\right)}{\partial \mathrm{pH}}\right)}_{E}$$

These numbers are consistent with those reported for the Co‐based perovskite OER catalysts which also utilized the LOM mechanism.^[^ ^23^, ^56^ ^]^ Of importance, the proton reaction order increased with increasing Sr‐deficiency level, indicating that the LOM pathway became more preferable for the perovskite samples with more Sr deficiencies.^[^ ^22^, ^23^ ^]^

Figure 2f compares the Tafel plots of the LaSr2−3*z* samples constructed using data from both CV and CA measurements (see Figure S8, Supporting Information, for an example of the CA data). Interestingly, the OER currents obtained from CV were found to be consistently larger than those measured from CA, although such a difference can be very minor compared to the previous literature results.^[^ ^22^ ^]^ This observation can be justified by the partial contribution of oxygen intercalation, as mentioned earlier, to the OER current during the non‐steady‐state CV testing, while such contribution is almost negligible during the steady‐state CA testing.^[^ ^22^, ^52^ ^]^ For the accuracy of measurement of intrinsic activity, steady‐state measurements such as CA may provide a better choice. Nonetheless, the presence of oxygen intercalation provides additional support to the lattice‐oxygen participation in the LaSr2−3*z* series.^[^ ^21^, ^22^, ^23^ ^]^ In addition, the reduced Tafel slopes with increasing Sr‐deficiency level, obtained from either the CV or the CA data, suggests that a larger Sr‐deficiency favors the OER kinetics of LaSr2−3*z*. Previous research has suggested that tetramethylammonium cation can interact with the active oxygen species generated during the LOM‐based OER process, resulting in a partial inhibition of the OER.^[^ ^36^, ^57^, ^58^ ^]^ We also compared the OER activity of LaSr1.90 in 1 M KOH with that in 1 M tetramethylammonium hydroxide (TMAOH) (Figure 2g). A decrease in OER activity along with an increase in Tafel slope was observed in the case of TMAOH, evidencing again the likely occurrence of the LOM mechanism.

Taken together, the above results suggest the operation of the LOM mechanism during the alkaline OER on the Sr‐deficient LaSr2−3*z* perovskites, the extent to which correlates strongly with the OER performance. It is important to note that, compared to the doped perovskite systems reported earlier,^[^ ^21^, ^23^ ^]^ where the crystal structure and/or electronic structure can change with the doping amount, our Sr‐deficient perovskite system manifests barely any change in these factors other than a modification in the concentration of oxygen vacancies and consequently their mobility, thereby offering more solid evidence to the contribution of LOM in the OER.

**Figure 3:**
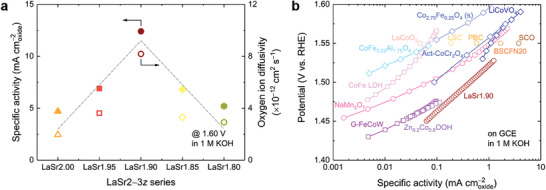
Optimized OER activity through maximizing the LOM participation and comparison with the state‐of‐the‐art OER catalysts. a) Comparison of OER activity at 1.60 V versus RHE in 1 M KOH and oxygen ion diffusivity of the LaSr2−3*z* series. The dash‐dot volcano lines are drawn to guide the eye to the overall trend of the data and are not linear regressions. b) Comparison of OER activity of the optimized sample LaSr1.90 with the state‐of‐the‐art catalysts, including multimetal (oxy)hydroxides (G‐FeCoW: gelled FeCoW oxyhydroxide,^[^ ^59^ ^]^ CoFe LDH: CoFe layered double hydroxide,^[^ ^59^ ^]^ and Zn_0.2_Co_0.8_OOH oxyhydroxide^[^ ^58^ ^]^), spinel oxides (CoFe_0.25_Al_1.75_O_4_,^[^ ^60^ ^]^ LiCoVO_4_,^[^ ^61^ ^]^ Act‐CoCr_2_O_4_: activated CoCr_2_O_4_,^[^ ^62^ ^]^ and Co_2.75_Fe_0.25_O_4_ (s): sulfurized Co_2.75_Fe_0.25_O_4_^[^ ^63^ ^]^), perovskite oxides (LaCoO_3_,^[^ ^22^ ^]^ LSC: La_0.5_Sr_0.5_CoO_3−_ *_*δ*_*,^[^ ^22^ ^]^ PBC: Pr_0.5_Ba_0.5_CoO_3−_ *_*δ*_*,^[^ ^22^ ^]^ SCO: SrCoO_3−_ *_*δ*_*,^[^ ^22^ ^]^ and BSCFN20: Ba_0.5_Sr_0.5_Co_0.6_Fe_0.2_Ni_0.2_O_3−_ *_*δ*_* ^[^ ^18^ ^]^), and other oxides (NaMn_3_O_7_^[^ ^64^ ^]^). All data were obtained in 1 M KOH using an RDE setup and all currents were normalized to the catalyst surface area to reflect the intrinsic OER activity.

The importance of lattice‐oxygen participation in the OER electrocatalysis was also supported by the catalytic performance for the cases with higher intentional Sr‐deficiency levels, i.e., LaSr1.85 and LaSr1.80 having intentional Sr‐deficiency levels of *z* = 0.050 and 0.067, respectively. As mentioned earlier, the pure‐phase structure was not obtained for LaSr1.85 and LaSr1.80 under the same synthesis conditions, and the extra deficiency introduced led to the formation of a (Co,Fe)_3_O_4_ minor phase instead (Figures S1 and S2, Supporting Information). Nonetheless, the surface chemical state of the transition‐metal cations in LaSr1.85 and LaSr1.80 was merely affected by the presence of the impurity, as evidenced by the almost identical XPS features to those of LaSr2.00, LaSr1.95, and LaSr1.90 (Figure S9, Supporting Information). However, the oxygen ion diffusivity underwent a decline with increasing intentional deficiency levels for LaSr1.85 and LaSr1.80 (Table S2, Supporting Information), likely caused by the blocking effect of the impurity phase that hinders charge transfer across the grains for oxygen ion diffusion.^[^ ^23^ ^]^ Electrochemical measurements show that intentionally increasing the deficiency level did not bring further activity enhancements, and the best OER performance was found for the LaSr1.90 sample (**Figure** **3**a), which had the highest A‐site cation deficiency while maintaining a single‐phase structure. Of significance, a strong correlation still holds for the whole LaSr2−3*z* series (3*z* = 0.00*–*0.20) between the OER specific activity and the oxygen ion diffusion rate (Figure 3a and Figure S10, Supporting Information). This correlation, together with the nearly unchanged transition metal electronic structure, therefore suggests that the increase in OER activity originated from the enhanced lattice‐oxygen participation, which can be facilely optimized through regulating the intentionally introduced A‐site cation deficiency amount. Markedly, the high intrinsic activity observed for the optimized LaSr1.90 sample was found to rank among the best records for nonprecious metal‐based OER catalysts, greater than or on par with the state‐of‐the‐art catalysts including (oxy)hydroxides,^[^ ^58^, ^59^ ^]^ spinel oxides,^[^ ^60^, ^61^, ^62^, ^63^ ^]^ perovskite oxides,^[^ ^18^, ^22^ ^]^ and other types of oxides,^[^ ^64^ ^]^ as presented in Figure 3b. Such a comparison again underlines the excellent OER activity of the LaSr1.90 perovskite catalyst enabled by the facile cation deficiency manipulation strategy.

**Figure 4:**
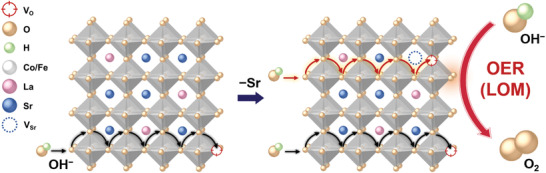
Schematic illustration showing how the introduction of A‐site Sr deficiency leads to the generation of additional oxygen vacancy and thus facilitates the OER catalysis through the LOM pathway. The oxygen ion diffusion process, as marked by the arrows, is illustrated to highlight its key role in promoting the LOM‐based OER process.

The above results highlight the advantage of A‐site cation deficiency manipulation as an effective way to promote the LOM‐based OER catalysis over perovskite oxides. As schematically shown in **Figure** **4**, by introducing A‐site Sr deficiency, an increased number of oxygen vacancies are created, leading to significant enhancements in the bulk oxygen mobility, which could enable fast replenishment of active lattice‐oxygen sites upon their evolution under the LOM scheme and hence result in improved OER performance.^[^ ^23^, ^52^ ^]^

### 2.3 Practical Applications in OER‐Related Energy Devices

**Figure 5:**
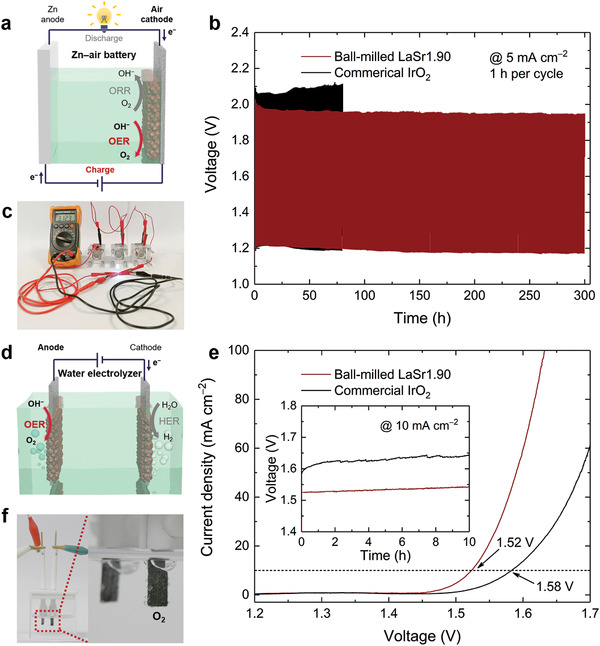
Evaluation of the electrochemical performance of the ball‐milled LaSr1.90 sample in a rechargeable Zn–air battery and a water electrolyzer. a) Schematic illustration of a rechargeable Zn–air battery. ORR: oxygen reduction reaction. b) Galvanostatic charge/discharge profiles of Zn–air batteries using the ball‐milled LaSr1.90 and the commercial IrO_2_ catalysts at 5 mA cm^−2^ (electrolyte: 6 M KOH + 0.2 M zinc acetate). c) Photograph showing how three Zn–air batteries assembled in series could power a light‐emitting diode. d) Schematic illustration of a water electrolyzer. HER: hydrogen evolution reaction. e) Polarization curves of water electrolyzers using the ball‐milled LaSr1.90 and the commercial IrO_2_, respectively, as anode catalyst and commercial 20 wt% Pt/C as cathode catalyst (electrolyte: 1 M KOH). Inset shows the chronopotentiometry curves at 10 mA cm^−2^. f) Photograph showing how the water electrolyzer could continuously generate gas bubbles during the stability test.

The OER occurs during the charging process of a rechargeable Zn‐air battery and is also the anodic half‐reaction of a water electrolyzer, which holds key to achieving high efficiencies for these energy devices.^[^ ^2^, ^3^, ^4^, ^5^ ^]^ Inspired by the outstanding OER activity of the optimized LaSr1.90 sample as discussed in previous investigations, we further evaluated its practical applications in rechargeable Zn‐air batteries and water electrolyzers using home‐made test models schematically illustrated in **Figure** **5**a,d. To achieve better performance in these energy devices, the pristine LaSr1.90 was subjected to ball‐milling to increase the number of active sites exposed as an effort to improve its electrode activity.^[^ ^13^ ^]^ In addition, commercial IrO_2_ was selected as a control. RDE studies showed that the pristine LaSr1.90 exhibited higher OER electrode activity compared to IrO_2_, which was further enhanced upon the ball‐milling treatment (Figure S11, Supporting Information). Figure 5b displays the galvanostatic charge/discharge profiles of Zn‐air batteries using the ball‐milled LaSr1.90 and IrO_2_, respectively, as the air cathode catalyst. To deliver a current density of 5 mA cm^−2^, the ball‐milled LaSr1.90 showed a charging voltage lower than IrO_2_ did (1.95 V vs. 2.10 V at 80 h), with remarkable stability in a prolonged cycling test for 300 h with no sign of decay. When coupled with a commercial Pt/C as cathode catalyst, the water electrolyzer with the ball‐milled LaSr1.90 as anode catalyst required a cell voltage of 1.52 V to drive a 10 mA cm^−2^ current density, much smaller than that with the IrO_2_ catalyst (1.58 V) (Figure 5e). Besides, more stable water electrolysis performance was also demonstrated by steady chronopotentiometric testing for 10 h (Figure 5e inset). In addition, Figure 5c shows that three Zn‐air batteries assembled in series could power a light‐emitting diode and Figure 5f shows that the water electrolyzer could continuously generate gas bubbles during the stability test, both showcasing the great potential of the cation‐deficient LaSr1.90 for commercial utilization in practical energy applications.

## 3 Conclusion

To summarize, we have designed in this work an A‐site Sr‐deficient perovskite system that catalyzes the OER more efficiently as more Sr deficiencies are introduced. Importantly, the introduction of Sr‐deficiency was found to modify solely the oxygen vacancy concentration and consequently the capability to allow for oxygen mobility, without impacting the material's crystal structure and/or electronic structure as encountered in perovskites doped by foreign elements. This finding, combined with the strong correlation between the OER activity and oxygen ion diffusivity, provides solid evidence supporting the key role of lattice‐oxygen participation in facilitating the OER over perovskite catalysts that utilize the LOM mechanism. Further, the OER activity can be facilely optimized by tuning the amount of A‐site deficiency that is intentionally introduced to the perovskite system, leading to the identification of highly efficient, cation‐deficient perovskite catalysts with great potential to be used in practical OER‐related energy applications such as rechargeable Zn‐air batteries and water electrolyzers. The fundamental understanding gained from this work can provide guidance for the design of catalyst materials with improved efficiencies for a wide variety of clean energy applications.

## Conflict of Interest

The authors declare no conflict of interest.

## Supporting information

Supporting information

## Data Availability

The data that support the findings of this study are available from the corresponding author upon reasonable request.
