# Supplementary material for: New Undisputed Evidence and Strategy for Enhanced Lattice‐Oxygen Participation of Perovskite Electrocatalyst through Cation Deficiency Manipulation
Source: Adv Sci (Weinh). 2022 Mar 20;9(14):2200530. doi: 10.1002/advs.202200530 (PMC9108636; doi:10.1002/advs.202200530)
Supplement: Supplementary file 1 — Supporting information [file ADVS-9-2200530-s001.pdf]

ADVANCED SCIENCE
Open Access

Supporting Information

for *Adv. Sci.*, DOI 10.1002/advs.202200530

New Undisputed Evidence and Strategy for Enhanced Lattice-Oxygen Participation of Perovskite Electrocatalyst through Cation Deficiency Manipulation

Xiaomin Xu, Yangli Pan, Yijun Zhong, Chenliang Shi, Daqin Guan, Lei Ge, Zhiwei Hu, Yi-Ying Chin, Hong-Ji Lin, Chien-Te Chen, Hao Wang, San Ping Jiang and Zongping Shao**

Supporting Information

New undisputed evidence and strategy for enhanced lattice-oxygen participation of perovskite electrocatalyst through cation deficiency manipulation

Xiaomin Xu, Yangli Pan, Yijun Zhong, Chenliang Shi, Daqin Guan, Lei Ge, Zhiwei Hu, Yi-Ying Chin, Hong-Ji Lin, Chien-Te Chen, Hao Wang, San Ping Jiang, Zongping Shao**

I. Experimental methods

Materials synthesis

The A-site Sr-deficient $La_{1/3}Sr_{(2-3z)/3}Co_{0.5}Fe_{0.5}O_{3-\delta}$ samples (or $LaSr_{(2-3z)}Co_{1.5}Fe_{1.5}O_{9-3\delta}$, denoted LaSr2−3*z*) were synthesized using a sol–gel method, as described in detail elsewhere.[1] All chemicals were of analytical grade and were used as received. The nitrates of La, Sr, Co, and Fe (Sigma-Aldrich) were adopted as raw materials and were dissolved in water and then chelated with complexing agents of citric acid (CA) (Chem-Supply Pty Ltd) and ethylenediaminetetraacetic acid (EDTA) (Sigma-Aldrich) at a metal-to-CA-to-EDTA ratio of 1:2:1, with the solution pH adjusted to about 6 to 8 by addition of an ammonia solution (Chem-Supply Pty Ltd). The mixed solution then underwent continuous stirring at a temperature of about 150 °C, resulting in evaporation of water and eventually the formation of a clear gel. The gel was heat-treated in a furnace at 250 °C for 5 h to obtain a solid precursor, which was then calcined in static air at a furnace temperature of 1050 °C for 10 h to yield the final products.

Characterizations

The room-temperature XRD analysis was conducted on an Aeris powder X-ray diffractometer with Co Kα radiation (wavelength: 1.7903 Å). The XRD data for the sample LaSr1.90 were also collected on a Bruker D8 Advance diffractometer with Cu Kα radiation (wavelength: 1.5418 Å), which were further analyzed by Rietveld refinement using the TOPAS 5 software. High-resolution transmission electron microscopy (HR-TEM) imaging was performed on a FEI Titan G2 80-200 instrument which operated at 200 kV. The Raman spectra were collected by a Raman spectrometer (Horiba LabRAM HR Evolution) using a 532-nm laser. XPS was performed on a Kratos Axis Ultra DLD spectrometer with Al Kα irradiation. The binding energy scale was calibrated to the C 1s peak for adventitious carbon (at 284.8 eV). Soft XAS data of the Co L_3-edge and Fe L_3-edge were collected in the total electron yield mode (TEY)

at the TLS BL11A beamline of the National Synchrotron Radiation Research Center (NSRRC) in Taiwan. CoO and Fe_2O_3 single crystals were measured simultaneously to calibrate the photon energy scales. The average B-site oxidation state and oxygen vacancy amount were estimated by iodometric titrations, following the referenced procedure.[2] In brief, about 0.1 g of perovskite sample was dissolved in a hydrochloride solution (70 vol% HCl (37 wt.%) and 30 vol% H_2O) containing excess potassium iodide (KI) in a flask, which was then titrated with a solution of 0.02 M sodium thiosulfate ($Na_2S_2O_3$). Starch was introduced to the solution to indicate the end-point of the titration. The average B-site oxidation state was calculated based on the amount of titrated $Na_2S_2O_3$ solution, and the oxygen vacancy amount was further deduced according to the charge-neutrality principle. BET surface area (S_{BET}, in $m^2\ g^{-1}$) was acquired by running nitrogen adsorption/desorption tests on a Micromeritics TriStar II *PLUS* instrument at −196 °C (i.e., nitrogen boiling point).

Electrochemical tests

The evaluation of the A-site Sr-deficient LaSr2−3*z* as the OER catalysts was carried out using a rotating disk electrode (RDE)-based methodology as reported in our previous work.[3] The perovskite powder was mixed with conductive Super P® carbon (Alfa Aesar) at a 5:1 mass ratio and then dispersed in a mixture of absolute ethanol and 5 wt% Nafion® 117 solution (Sigma-Aldrich) at a 9:1 volume ratio to obtain a catalyst ink, which, upon ultrasonic treatment for 1 h, was deposited on a glassy carbon electrode (5 mm diameter) with a mass loading of 0.255 $mg_{oxide}\ cm^{-2}$ to serve as the working electrode. The electrochemical cell, reference electrode (Ag/AgCl), and counter electrode (Pt wire) were all purchased from Pine Research Instrumentation. The electrochemical data were collected on a Biologic VSP Potentiostat. Cyclic voltammetry (CV) was performed at a scan rate of 10 $mV\ s^{-1}$ to analyze the kinetic currents in freshly prepared KOH solutions with varying pH. The same CV measurement was also performed in a freshly prepared 1 M tetramethylammonium hydroxide (TMAOH) solution. Steady-state currents were also collected by multistep chronoamperometry (CA) in a potential range of about 0.46–54 V vs. Ag/AgCl at a 0.01 V increment. During these measurements, the working electrode was rotated at 2,400 rpm to readily remove oxygen bubbles generated at the catalyst surface. The data processing follows the same procedure as described earlier.[3] Briefly, the current was averaged and normalized by BET surface area to obtain the specific activity, and the potential was *iR*-corrected and converted to the RHE scale, whose difference with respect to the equilibrium potential (1.229 V vs. RHE) was calculated to be the overpotential.

Oxygen ion diffusivity measurements

The oxygen ion diffusivity measurements were performed in an Ar-saturated 6 M KOH solution using combined CV and CA techniques. The same working and counter electrodes were applied, while Hg/HgO was used as the reference electrode due to the strongly alkaline condition. CV scan was taken at 20 mV s^{-1} with the working electrode held stationary, followed by CA test performed at a potential 50 mV more positive of $E_{1/2}$ (defined as the potential halfway between the redox peaks found in the CV data) with the working electrode rotated at 2,400 rpm to eliminate any electrolyte-based mass-transfer effect. The oxygen ion diffusion coefficient (D_O) was calculated according to the equation $\lambda = a(D_O t)^{-1/2}$ by adopting a bounded 3D diffusion model.[4] Here, $t^{-1/2}$ was obtained as the intercept (at i = 0) for the linear fitting of the i vs. $t^{-1/2}$ plot (i.e., current as a function of the inverse square root of time) from the CA measurement, a, radius of the perovskite particle, was estimated using the relation of $S_{BET} = 6/(2a\rho)$ based on a spherical geometry approximation, where S_{BET} is the BET surface area and ρ is the theoretical density obtained from XRD analysis, and λ, a dimensionless shape factor, was assumed to be 2, representative of a rounded parallelepiped, halfway between the values for a cube (λ = 2.26) and a sphere (λ = 1.77).[3]

Assembling and testing of water electrolyzers and Zn–air batteries

To assess the practical use of the optimized LaSr1.90 catalyst, a ball-milling treatment was first performed at 400 rpm for 1 h using a high-energy ball mill (Planetary Mono Mill, Pulverisette 6, Fritsch) to increase the electrode activity. A home-made, two-electrode water electrolyzer device was employed to measure the overall water splitting performance. A catalyst ink made of ball-milled LaSr1.90 was prepared following the same procedure as mentioned in the RDE testing methodology, which was deposited on a 0.5 cm × 1 cm carbon paper (Sigracet 39 AA, Fuel Cell Store) with a perovskite mass of 1 mg to serve as the anode. Commercial product of 20 wt% Pt/C (HiSPEC® 3000, Alfa Aesar), prepared into a catalyst ink using the same method, was deposited on the same carbon paper with a Pt/C mass of 1 mg to serve as the cathode. Polarization curves were collected on a CHI760E bipotentiostat (CH Instruments, Inc.) using linear sweep voltammetry (LSV) at a scan rate of 5 mV s^{-1} in a 1 M KOH electrolyte. Chronopotentiometry curves were recorded at 10 mA cm^{-2} for 10 h to assess the stability. A Zn–air battery was assembled with an air cathode, a zinc plate anode, and an electrolyte made of 6 M KOH and 0.2 M zinc acetate in a home-made test model. The ball-milled LaSr1.90 was mixed with carbon nanotubes (Sigma-Aldrich) at a 2:1 mass ratio and dispersed in a mixture of absolute ethanol and 5 wt% Nafion® 117 solution at a 9:1 volume ratio to obtain a catalyst ink, which was then deposited on a gas diffusion layer (AvCarb P75T) with an effective area of 0.5 cm^2 to yield the air cathode with a catalyst

loading of 3 mg cm^{-2}. Galvanostatic charge/discharge profiles of the Zn–air battery were obtained at 5 mA cm^{-2} using a LANHE CT2001A battery test system. For comparison, commercial IrO_2 (Sigma-Aldrich) was applied as a control sample and evaluated using the same methods mentioned above and in the same water electrolyzer and Zn–air battery devices.

II. Supplementary results

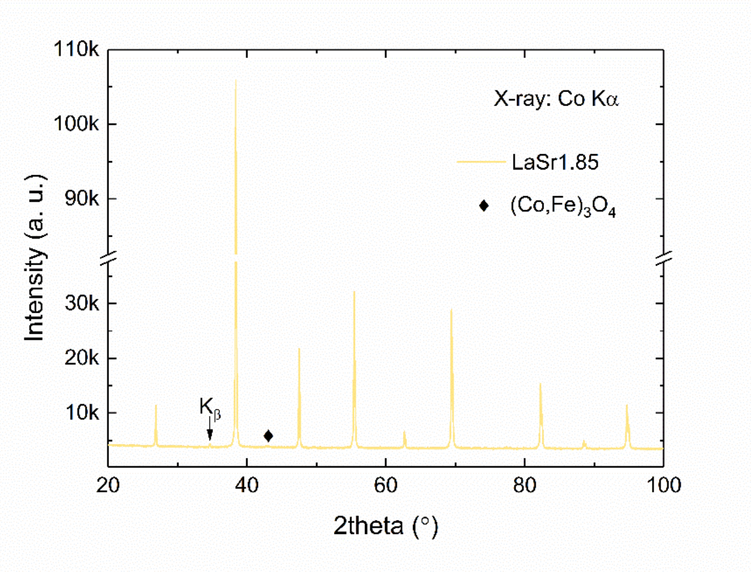

Figure S1. XRD data of $LaSr_{1.85}Co_{1.5}Fe_{1.5}O_{9-3\delta}$ (denoted LaSr1.85) showing the formation of a minor impurity phase $(Co,Fe)_3O_4$ (as marked by the solid diamond).

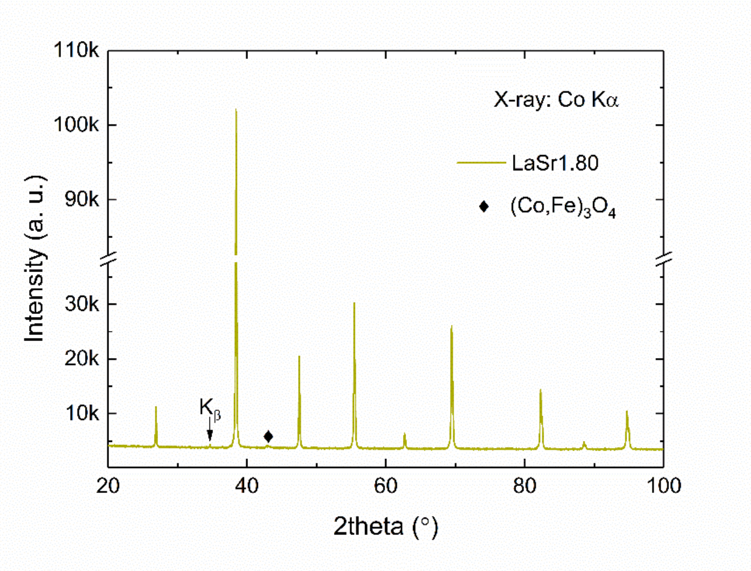

Figure S2. XRD data of $LaSr_{1.80}Co_{1.5}Fe_{1.5}O_{9-3\delta}$ (denoted LaSr1.80) showing the formation of a minor impurity phase $(Co,Fe)_3O_4$ (as marked by the solid diamond).

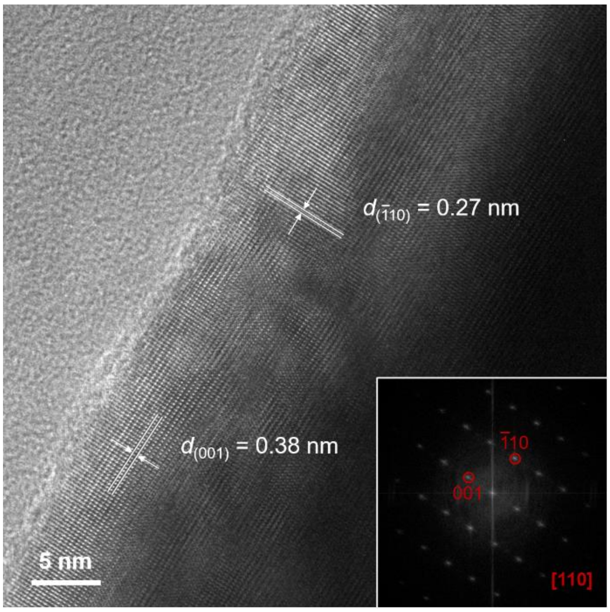

Figure S3. High-resolution transmission electron microscopy (HR-TEM) image of LaSr1.90, with the corresponding fast Fourier transformed (FFT) pattern shown in the inset.

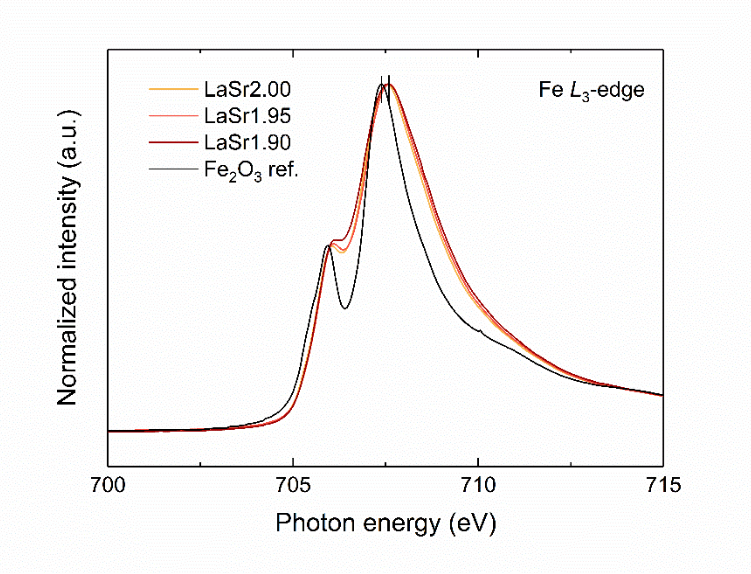

Figure S4. Fe *L*-edge XAS spectra of LaSr2.00, LaSr1.95, and LaSr1.90 (collected in the TEY mode). Inset shows an enlargement of Fe L_3-edge spectra.

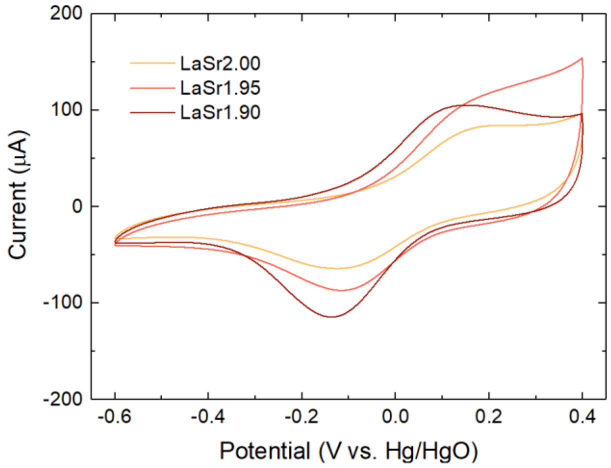

Figure S5. CV curves of LaSr2.00, LaSr1.95, and LaSr1.90 obtained in Ar-saturated 6 M KOH, where redox peaks indicate the electrochemical intercalation and de-intercalation of oxygen ions.

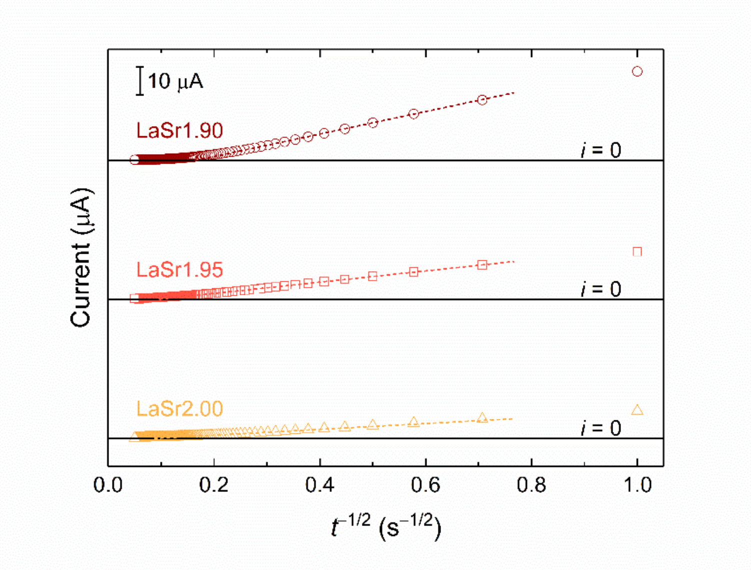

Figure S6. CA data of LaSr2.00, LaSr1.95, and LaSr1.90, where current is plotted as a function of the inverse square root of time (i vs. $t^{-1/2}$). The linear portion was fitted to obtain the intercept with the $t^{-1/2}$ axis (at $i = 0$).

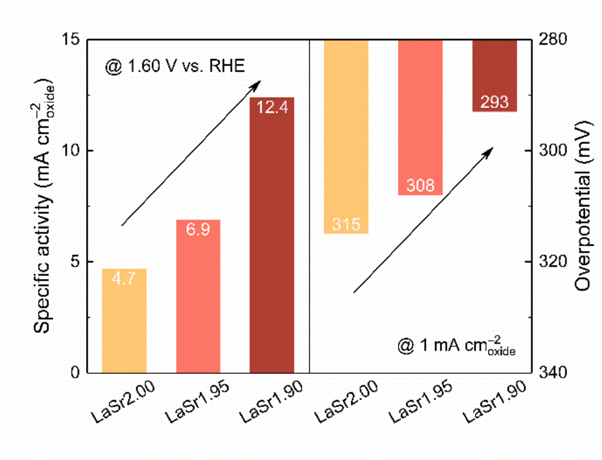

Figure S7. Comparison of OER activity in terms of the current density at a select potential of 1.60 V vs. RHE and the overpotential at a given current density of 1 mA cm^{-2}_{oxide}. The electrochemical data were obtained by performing CV measurements in oxygen-saturated 1 M KOH electrolyte under ambient temperature.

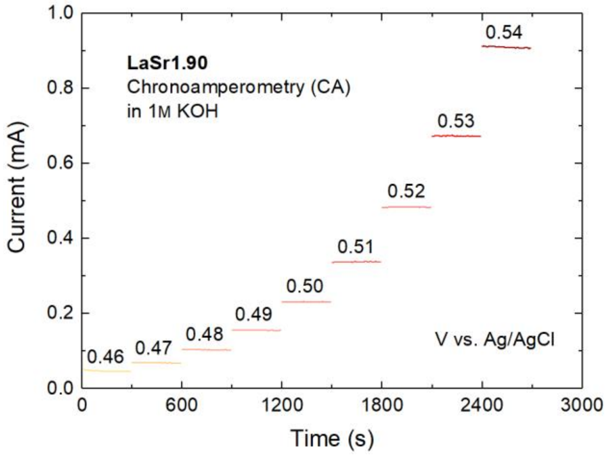

Figure S8. CA data of LaSr1.90 collected in 1 M KOH under varying potentials (V vs. Ag/AgCl).

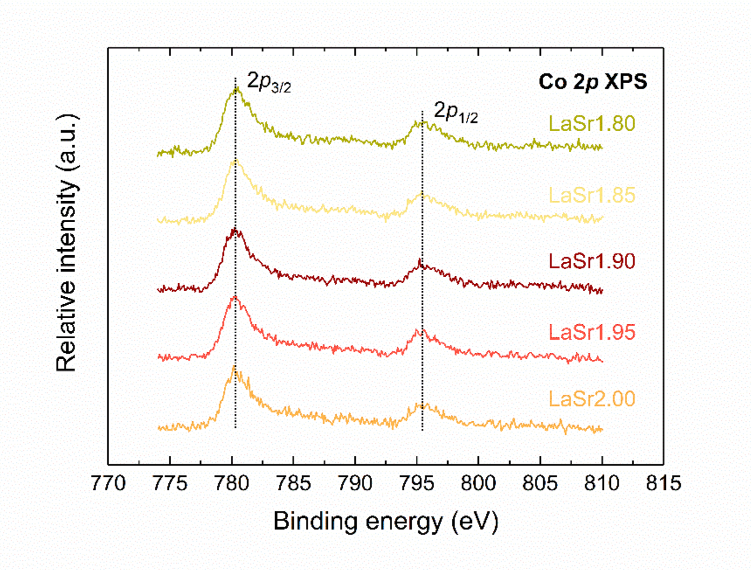

Figure S9. Co 2*p* XPS spectra of LaSr2.00, LaSr1.95, LaSr1.90, LaSr1.85, and LaSr1.80.

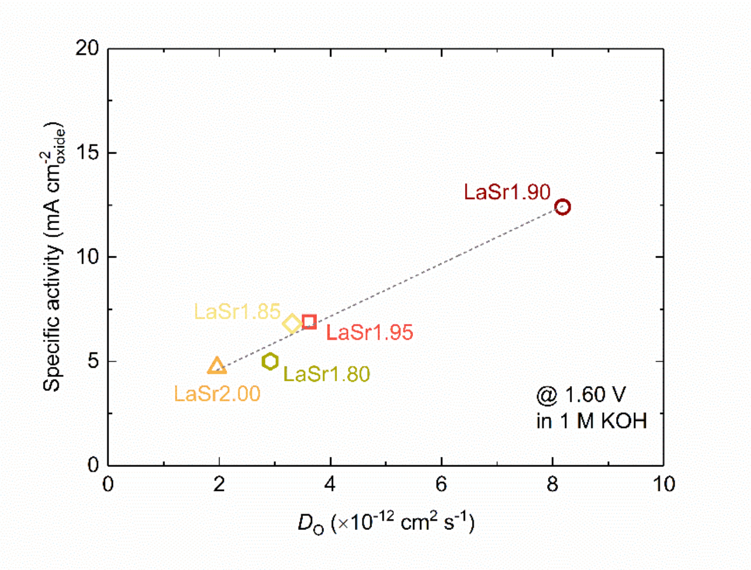

Figure S10. Correlation of intrinsic OER activity in 1 M KOH at 1.60 V vs. RHE with the oxygen ion diffusion coefficient (D_O) for the whole LaSr2−3*z* series (3*z* = 0.00 – 0.20).

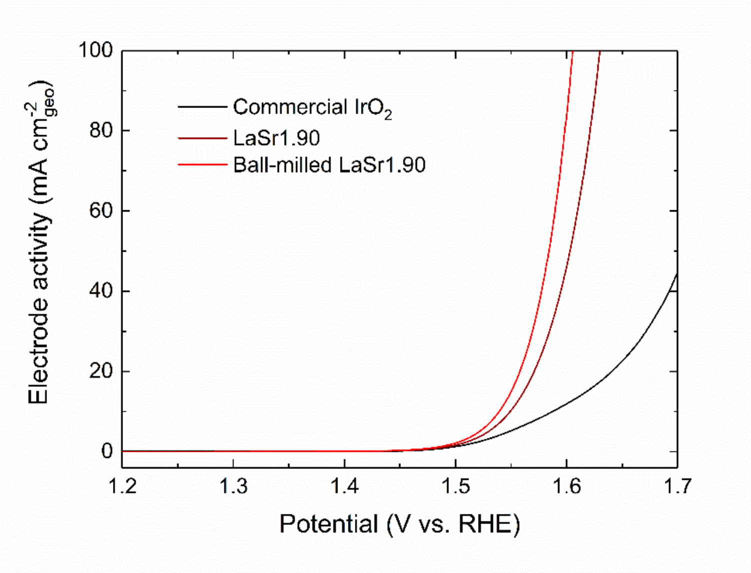

Figure S11. Electrode activity of the pristine LaSr1.90 and ball-milled LaSr1.90 in comparison to the commercial IrO_2 (electrolyte: 1 M KOH). The ball-milled LaSr1.90 and the commercial IrO_2 were deposited on a glassy carbon electrode with a mass loading of 0.255 mg_{oxide} cm^{-2} using the same catalyst ink preparation method as the pristine LaSr1.90. The OER current was normalized to the geometric surface area of the glassy carbon electrode to obtain the electrode activity (in mA cm_{geo}^{-2}).

Table S1. The average B-site oxidation state and oxygen vacancy concentration (δ) of the Sr-deficient LaSr2−*z* perovskites, as estimated from the iodometric titration method.

Perovskite	Nominal composition [$La_{1/3}Sr_{(2-z)/3}Co_{0.5}Fe_{0.5}O_{3-\delta}$]	δ	Average B-site oxidation state
LaSr2.00	$La_{1/3}Sr_{2.00/3}Co_{0.5}Fe_{0.5}O_{3-\delta}$	0.011	3.645
LaSr1.95	$La_{1/3}Sr_{1.95/3}Co_{0.5}Fe_{0.5}O_{3-\delta}$	0.023	3.654
LaSr1.90	$La_{1/3}Sr_{1.90/3}Co_{0.5}Fe_{0.5}O_{3-\delta}$	0.042	3.649

Table S2. Oxygen ion diffusion coefficient (D_O) of the Sr-deficient LaSr2−*z* perovskites.

Perovskite	ρ [g cm^{-3}]	S_{BET} [m^2 g^{-1}]	a [nm]	λ	$t^{-1/2}_{(i=0)}$ [s$^{-1/2}$]	D_O [×10^{-12} cm^2 s^{-1}]
LaSr2.00	6.0766	1.41	351	2	0.0798	1.96
LaSr1.95	6.0501	1.24	400	2	0.0951	3.62
LaSr1.90	6.0262	1.47	339	2	0.1687	8.18
LaSr1.85	5.9758	1.31	384	2	0.0947	3.31
LaSr1.80	5.9431	1.18	428	2	0.0799	2.92

Table S3. BET surface areas (S_{BET}) of the Sr-deficient LaSr2−3*z* perovskites.

Perovskite	S_{BET} [m^2 g^{-1}]
LaSr2.00	1.41
LaSr1.95	1.24
LaSr1.90	1.47
LaSr1.85	1.31
LaSr1.80	1.18
Ball-milled LaSr1.90	5.38

III. Supplementary references

[1] X. Xu, Y. Pan, L. Ge, Y. Chen, X. Mao, D. Guan, M. Li, Y. Zhong, Z. Hu, V. K. Peterson, M. Saunders, C.-T. Chen, H. Zhang, R. Ran, A. Du, H. Wang, S. P. Jiang, W. Zhou, Z. Shao, *Small* **2021**, *17*, 2101573.

[2] Q. Sun, Z. Dai, Z. Zhang, Z. Chen, H. Lin, Y. Gao, D. Chen. *J. Power Sources* **2019**, *427*, 194.

[3] Y. Pan, X. Xu, Y. Zhong, L. Ge, Y. Chen, J.-P. M. Veder, D. Guan, R. O'Hayre, M. Li, G. Wang, H. Wang, W. Zhou, Z. Shao, *Nat. Commun.* **2020**, *11*, 2002.

[4] F. R. Van Buren, G. H. J. Broers, A. J. Bouman, C. Boesveld, *J. Electroanal. Chem. Interfacial Electrochem.* **1978**, *87*, 389.
